# Site-specific transgene integration in chimeric antigen receptor (CAR) T cell therapies

**DOI:** 10.1186/s40364-023-00509-1

**Published:** 2023-07-04

**Authors:** Hamed Dabiri, Pooria Safarzadeh Kozani, Mahdi Habibi Anbouhi, Mohadeseh Mirzaee Godarzee, Mohammad Hossein Haddadi, Mohsen Basiri, Vahab Ziaei, Majid Sadeghizadeh, Ensiyeh Hajizadeh Saffar

**Affiliations:** 1 Department of Genetics, Faculty of Biological Sciences, Tarbiat Modares University, Tehran, Iran; 2 Department of Stem Cells and Developmental Biology, Cell Science Research Center, Royan Institute for Stem Cell Biology and Technology, ACECR, Tehran, Iran; 3 Department of Medical Biotechnology, Faculty of Medical Sciences, Tarbiat Modares University, Tehran, Iran; 4 National Cell Bank of Iran, Pasteur Institute of Iran, Tehran, Iran; 5 Clinical Microbiology Research Center, Ilam University of Medical Sciences, Ilam, Iran; 6 Department of Regenerative Medicine, Cell Science Research Center, Royan Institute for Stem Cell Biology and Technology, ACECR, Tehran, Iran; 7 Advanced Therapy Medicinal Product Technology Development Center (ATMP-TDC), Royan Institute for Stem Cell Biology and Technology, ACECR, Tehran, Iran

**Keywords:** Chimeric antigen receptor, Cancer immunotherapy, Genome-editing technologies, Retroviral vectors, Natural killer cells

## Abstract

Chimeric antigen receptor (CAR) T cells and natural killer (NK) cells are genetically engineered immune cells that can detect target antigens on the surface of target cells and eliminate them following adoptive transfer. Recent progress in CAR-based therapies has led to outstanding clinical success in certain patients with leukemias and lymphomas and offered therapeutic benefits to those resistant to conventional therapies. The universal approach to stable CAR transgene delivery into the T/NK cells is the use of viral particles. Such approaches mediate semi-random transgene insertions spanning the entire genome with a high preference for integration into sites surrounding highly-expressed genes and active loci. Regardless of the variable CAR expression level based on the integration site of the CAR transgene, foreign integrated DNA fragments may affect the neighboring endogenous genes and chromatin structure and potentially change a transduced T/NK cell behavior and function or even favor cellular transformation. In contrast, site-specific integration of CAR constructs using recent genome-editing technologies could overcome the limitations and disadvantages of universal random gene integration. Herein, we explain random and site-specific integration of CAR transgenes in CAR-T/NK cell therapies. Also, we tend to summarize the methods for site-specific integration as well as the clinical outcomes of certain gene disruptions or enhancements due to CAR transgene integration. Also, the advantages and limitations of using site-specific integration methods are discussed in this review. Ultimately, we will introduce the genomic safe harbor (GSH) standards and suggest some appropriate safety prospects for CAR integration in CAR-T/NK cell therapies.

## Introduction

The immune system has an exceptional ability to scan the body and eradicate malignant cells following their recognition [1]. Adoptive immunotherapy approaches utilize and improve the strength of the immune system for more specific detection and elimination of tumor cells [1]. Chimeric antigen receptors (CARs) are synthetic molecules that benefit from T-cell receptor (TCR) signaling and the specificity of monoclonal antibodies (mAbs) developed for redirecting immune cells against tumor cells of interest [2]. Today, CAR-T/natural killer (NK) cell therapy advances bring hope to patients with blood-based malignancies for recovery [2].

As of February 2022, the United States Food and Drug Administration (FDA) has endorsed six CAR T cell products for the treatment of certain cancer patients with blood-based cancers [3]. These autologous products, including *axicabtagene ciloleucel* (*Yescarta*), *tisagenlecleucel* (*Kymriah*), *brexucabtagene autoleucel* (*Tecartus*), and *lisocabtagene maraleucel* (*Breyanzi*), target CD19 as the target antigen, and have been approved for the treatment of subsets of patients with CD19-associated malignancies [4].

Moreover, in 2021 and 2022, the US FDA also gave approval to i*decabtagene vicleucel* (*Abecma*) and *ciltacabtagene autoleucel* (*Carvykti*) indicated for certain patients with multiple myeloma [4]. Of note, both of these products are redirected against the B-cell maturation antigen (BCMA) [4, 5]. Currently, evaluating different autologous and/or allogeneic CAR T cells that target different sets of antigens is a field of interest for researchers [4]. In the process of CAR-based therapeutics, the desired CAR construct is transferred into the target immune cells using viral particles, mRNAs, and transposons [6, 7].

Virus-based vectors are the most common approach to stable CAR gene expression in the T/NK cells [8]. These retroviral vectors include γ-retroviral vectors and lentiviral vectors, and they have been derived from murine leukemia viruses (MLVs) and HIV-1, respectively [8]. Such vectors mediate semi-random insertion of the CAR transgene into different genome sites (spanning the entire genome) with the preference of highly expressed genes and open chromatin loci [9].

Using non-viral methods is a cost-effective way of CAR T cell engineering [10]. In the past decades, the Sleeping Beauty transposons (SB), a non-viral method, have been developed that contain a construct of CAR gene and transposition elements that transfer into the target cells by cationic polymers or electroporation as the delivery system [10]. In this method, the transgenes can be integrated into genomic sites that are distant from highly expressed genes or from oncogenes [10]. However, there are safety concerns and uncertainties regarding the clinical applicability of such non-viral methods. For instance, in 2021, Micklethwaite and colleagues reported the results of a Phase I clinical investigation (ACTRN12617001579381) in which patients with B-cell malignancies underwent CD19-redirected CAR T cells for the development of which a piggyBac transposon method was employed, rather than viral vectors [11]. What came as a surprise was that two patients developed CAR T cell-related lymphoma following treatment, which were progressive as one of the patients eventually submitted to the disease [11]. Following in-depth analysis, it was demonstrated that the genes of the surrounding regions of the integrated transgene had elevated transcription rates which were mediated by the promoter of the integrated DNA fragment [11]. Moreover, as a high number of transgene copies and point mutations (not linked to the integration site) were documented, the transgene was not reported to be integrated into known oncogenes [11]. The findings of Micklethwaite and colleagues accentuate the fact that patients that undergo CAR T cell therapy developed by novel genetic engineering methods need to be closely and regularly monitored [11]. Furthermore, profound clinical investigations must be conducted to fully assess the safety and clinical feasibility of any given genetic engineering method utilized for the development of genetically manipulated therapeutics.

The integrated transgene(s) might influence the adjacent genes’ expression and/or chromatin structure [12], which might result in the perturbance of the engineered effector cell function or even drive them towards neoplasm [13]. The most common event is a gain-of-function mutation that acts *dominant* [13]. However, randomly inserted genes could be subject to positional effects and silencing, making their expression unreliable and/or unpredictable. For instance, centromeres and near telomeres are locations particularly susceptible to silencing of the inserted foreign genes [13].

Recently, various strategies have been developed to integrate a foreign DNA fragment into a specific location in the human genome, mainly based on the DNA repair mechanism [14]. In this way, rare-cutting endonucleases or ribonucleoproteins that are activated in response to DNA double-strand breaks (DSBs) are utilized. Emerging technologies, such as recombinant Adeno-Associated Viruses (rAAV), meganucleases, zinc finger nucleases (ZFN), transcription activator-like effector nucleases (TALEN), and CRISPR-Cas9, has enabled us to integrate DNA fragments of interest into desired sites in the human genome [4, 7]. Using homologous recombination and Adeno-Associated Viruses (AAVs) for efficient site-directed gene insertion for proper transfer and expression of CAR constructs in primary T/NK cells is an example in this regard [15]. However, the potential risks associated with the induction of DNA DSBs and repairs as well as the genome-wide specificity of artificial endonucleases require in-depth research or long-term patient follow-up [15].

Even though these tools have experienced remarkable progress, there is a critical question that has not yet been fully answered. To achieve the highest safety index and expression efficacy, where a CAR transgene must be introduced? Is it possible to integrate a CAR transgene into genes that are not only dispensable for cells but also their disruption promotes the tumoricidal functionality of CAR T/NK therapies? Of note, insertion into some genomic sites may be suitable for somatic cells but not for T/NK cells [16]. Moreover, the effect of the newly integrated DNA on adjacent genes should be fully evaluated and understood in CAR T/NK cells [17]. So, the main issue is which site would be safe and suitable? In this review, we aim to address these questions by exploring the findings that have been gathered by researchers in the context of gene engineering.

## Advantages of site-specific integration in CAR-T/NK cell therapies

Retrovirus (RV) and lentivirus (LV) derived-vectors are known as popular vectors for CAR transgene delivery into T/NK cells [8, 18]. It has recently been recognized that they might induce immunoreaction and insertional mutagenesis [19]. Ruella et al. reported that the CAR DNA was accidentally incorporated into the genome of a single leukemic B cell in the process of CAR T cell manufacturing, while they observed that the disease relapse 9 months after CD19-redirected CAR T cell treatment [19]. Such incidents demonstrate the risks associated with random gene insertion in clinical settings. The advantages of both RV and LV vectors are their high gene transfer efficiency and stable CAR expression [19]. Although, both systems have been shown to be safe in intensive biosafety testing for recent CAR T cell therapies, this safety issue still remains a concern for long-term use and a variety of other gene therapy platforms [19].

LV- and RV-mediated random gene integration into the genome is unpredictable [20]. This may lead to oncogenesis, fluctuating CAR expression levels, and gene silencing [21]. A random integration causes substantial variations in the CAR expression level in a batch of CAR T cells because of the different copy numbers per cell. Also, there are some other drawbacks [21], such as autoimmune disorders induced due to permanent expression of CARs [22]; therefore, it is necessary to establish a site-specific vector ensuring insertion of CARs into genomic safe harbors (GSHs), which may contribute to safe, long-term, and dynamic CAR expression [21].

**Fig. 1:**
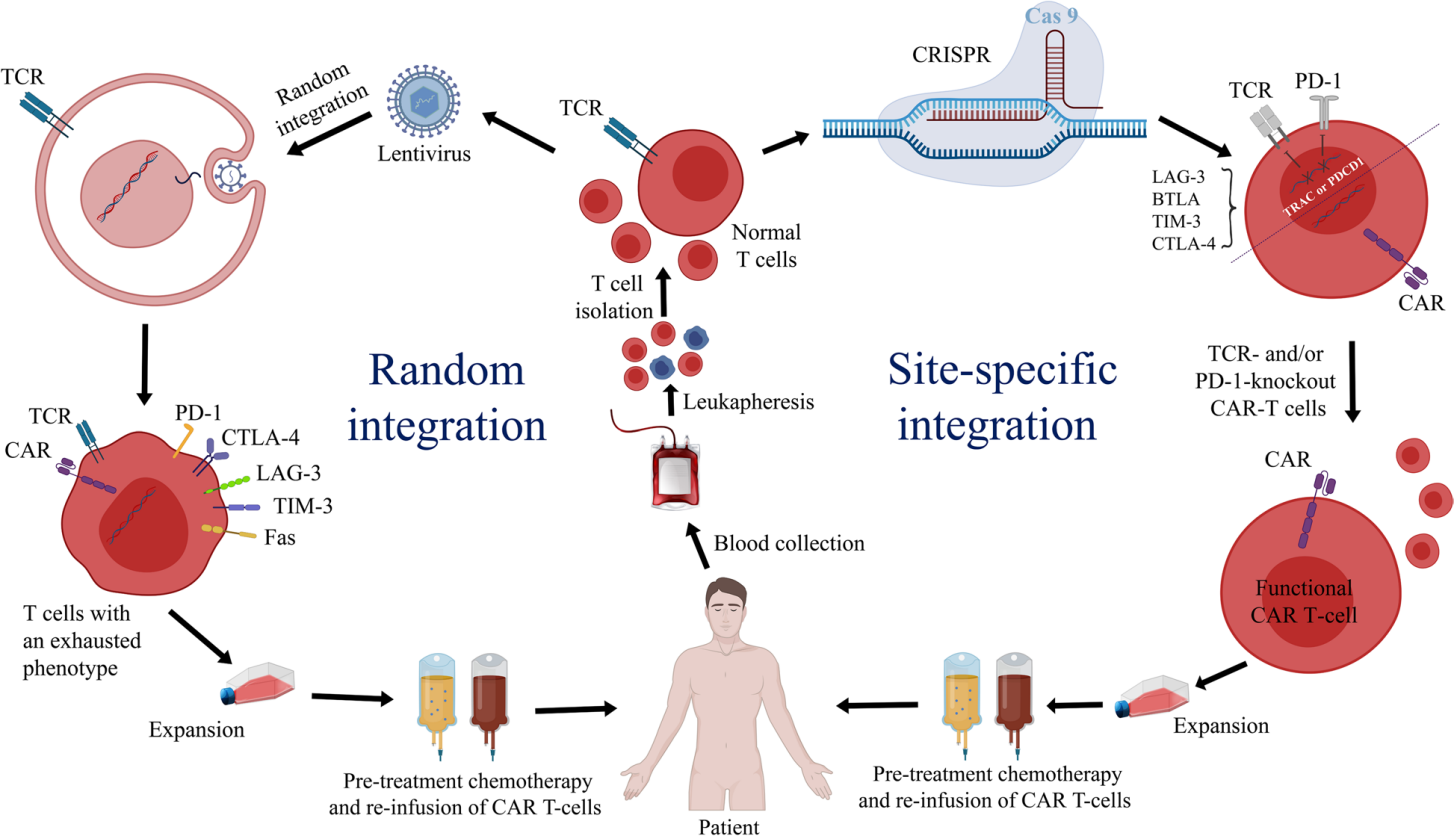
A comparison between random integration and site-specific integration in CAR T cell therapy. Random integration: random integration with viral particles usually includes the utilization of strong viral or non-viral promoters (CMV and EF1α, respectively) that constantly express the CAR transgene if integrated into highly expressed regions. This event is occasionally accompanied by strong exogenous gene expression by normal T/NK cells as well as antigen-independent tonic signaling due to the CAR clustering phenomenon. The pressure and tonic signaling result in the exhaustion of CAR T cells with less central memory phenotype and consequently poor clinical outcomes. Site-specific integration: site-specific integration of the CAR transgene under the active promoters of T/NK cells (such as TCRα) not only does not impose any external pressure but it also allows the CAR transgene to use the corresponding regulatory factors other than the promoter (such as the enhancer/silencer) and also enables the secondary structure of chromatin to regulate CAR expression as a dynamic phenomenon. Therefore, long-lasting central memory T cells with low exhaustion phenotype and high antitumor functionality can be expanded and employed to eradicate tumor cells

In the context of gene therapy, the controlled integration of foreign DNA into the genome is an obvious advantage, which can circumvent the dangers associated with a random transgene integration (Fig. 1) [6]. Another advantage in this regard is disrupting certain genes that can promote the therapeutic efficacy of CAR T/NK cells [6]. All these advantages are achieved with site-specific integration of exogenous DNA into the genome [23]. Moreover, it has been demonstrated that the efficacy and persistence of CAR T/NK cells may be promoted by the amplification of certain gene expression level or signaling pathways*,* all of which could be influenced by the regulatory elements of the CAR construct on the adjacent genes.

Generally, tonic signaling is becoming more well-recognized as a problem that can result in the poor antitumor efficacy, diminished survival, and decreased persistence of CAR T/NK cells in vivo [5, 6, 24]. This event can also promote T cell anergy, exhaustion, and activation-induced cell death [24]. So far, various efforts have been made to reduce the tonic signaling of CAR T cells which include substitution of scFv-based targeting domains, adjusting the hinge/spacer, optimal selection of the transmembrane domain and/or costimulatory intracellular domains, and controlling CAR expression [5, 18, 24]. There is a hypothesis that unconstrained CAR expression may result in tonic signaling. Based on this, it was hypothesized that the intermittent use of a site-specific insertion by genome-editing technologies could mitigate these negative effects and improve antitumor efficacy [24]. One of the most effective techniques in this respect has been the expression of the CAR transgene under the control of the TRAC promoter and its regulatory components [25]. Non-viral gene editing results in uniform CAR expression which averts tonic CAR signaling and establishes an effective internalization and re-expression of the CAR following a single or repeated exposure to antigen, thereby delaying effector T cell differentiation and exhaustion [25]. Hopefully, additional target genes besides TRAC may emerge in this regard in the near future.

### T cell advantages

In the context of T cells, site-specific CAR gene insertion enables an allogeneic CAR T cell production owing to the disruption of genes that drive the ability of CAR T cells to mediate *graft-versus-host disease* (GvHD) [26, 27]. Such genes can also be knocked out using genome-editing tools. Allogeneic CAR T cell therapies have several advantages over their autologous counterparts [28]. First, T cells can be obtained from a healthy donor and then screened for the desired phenotypic characteristics and an acceptable CD4:CD8 ratio [27–29]. Second, in an autologous setting, the most important limitation is the low number of the patient’s T cells at the time of apheresis due to the previous chemotherapeutic regimens which leads to difficulties in the process of CAR T cell manufacturing [27, 28]. Moreover, the ability to prepare appropriate starting materials for an allogeneic CAR T cell product enables superior control of the procedure, generation of more reliable and homogeneous products, and the availability of products without batch-to-batch variation [28]. Third, it should be feasible to generate plenty of therapeutic CAR T cell doses from a single *good manufacturing practice* (GMP) run; therefore, allogeneic CAR T cells have a timing advantage as the living drug would be available “*off-the-shelf*”; thus obviating the need for patient leukapheresis and minimizing hospitalization period [28].

A site-specific integration enables the production of more effective CAR T cell therapies against solid tumors due to the disruption of genes involved in the immunosuppression pathways [30]. Solid tumors are considerably more difficult to eliminate because of the complex inhibitory factors in the tumor microenvironment (TME) [30–34]. An immune escape due to the suppression of activated cytotoxic T cells is another major factor that happens through the interaction of T cell inhibitory receptors with their ligands on solid tumor cells, such as programmed cell death-1 (PD-1) and cytotoxic T-lymphocyte antigen 4 (CTLA-4) [35].

In addition, the function of T cells is impaired by exhaustion, particularly in patients with chronic infections and different cancers, during which T cells are exposed to persistent antigen and/or inflammatory signals [36, 37]. The effector functions of exhausted immune cells diminish because of the expression of multiple inhibitory receptors and altered transcriptional profiles. In particular, PD-1, CTLA-4, TIM-3, and LAG-3 have been shown to play particular roles in T cell exhaustion [38]. Reversing T cell exhaustion by blocking PD-1 or CTLA-4 checkpoint has shown promising clinical outcomes [38]. Thus, the generation of T cells resistant to multiple inhibitory pathways is expected to improve the function of CAR T cell therapy of solid tumors, a task that can be accomplished by taking advantage of a site-specific integration.

The tumor necrosis factor α (TNF-α) family of death receptors induces immune cell apoptosis which negatively affects the outcome of immunotherapies [39]. Among them, the *Fas* receptor is a known immunotherapy obstacle as reports have indicated a diminished CAR T cell activity due to a phenomenon called Fas-FasL-dependent activation-induced cell death (AICD) [39]. Thus, targeting Fas-induced cell death using gene disruption or a gene-editing approach might lead to the improvement of CAR-T cell function [39].

### NK cell advantages

NK cells have become a popular immunotherapy source that can be collected from unrelated donors since they do not mediate graft-versus-host, which can also be investigated as "off-the-shelf" adoptive products. Similar to T cells, a reduced NK cell activity can potentially be caused by changes in the NK cell receptor repertoire and the TME ligand expression level. As a result, addressing the receptor repertoire, specifically by reducing NK cells inhibitory signals, is expected to improve their anti-tumor efficacy. The combination of site-specific CAR integration and gene knock-out in NK cells offers new possibilities for advanced CAR-NK cells [40].

Blocking the inhibitory signals of NK cells might increase the effectiveness of NK-based cancer treatment. In this regard, Pomeroy et al. used a CRISPR/Cas9 system to knock out inhibitory signaling molecules in human NK cells. They showed successful knockdown of the ADAM17 and PD-1 genes, as contributors to the NK cell functions. They reported that these gene-edited NK cells had dramatically increased activity, cytokine secretion, and cytotoxicity against tumor cells. They were also able to increase cells to clinically relevant numbers while maintaining activity [41].

In cancer immunotherapy, the patient's immune system recognizes and rejects the infused NK cells, limits their life span in vivo*,* and eliminates the prospect of multiple infusions. Hoerster et al. used a genome-editing strategy and improved the lentiviral transduction procedure in primary human NK cells to render them resistant to the CD8^+^ T cell responses of the recipients [42]. They coexpressed a single-chain HLA-E molecule after knocking down the surface expression of the HLA class I molecules via the B2M gene targeting [42]. They used CRISPR/Cas9 to inhibit the NK cell fratricide of B2M-knockout (KO) cells via "missing self"-induced lysis [42]. Importantly, in terms of phenotypic and natural cytotoxicity against several AML cell lines, these genetically edited NK cells were functionally identical to their unmodified counterparts [42]. This research shows that genome editing in primary allogeneic NK cells can reduce the recognition and killing of these cells by mismatched T cells, which is a prerequisite for using non-HLA-matched primary human NK cells as readily available "off-the-shelf" immune effectors for a variety of immunotherapeutic purposes [42]. The combination of site-specific CAR integration and gene knock-out in NK cells offers up new possibilities for advanced primary CAR-NK cells [42].

## Specific gene editing improves the phenotypic characteristics or promotes the therapeutic efficacy of CAR T/NK cells

Fraietta et al. expanded an individual clone of CAR T cells derived from a patient with chronic lymphocytic leukemia (CLL) and found a dominant population of the infused CD19-redirected CAR-T cells [43]. Following CAR T cell treatment, antitumor efficacy was evident and the patient achieved complete remission (CR) after long-term follow-up evaluation of more than 4.2 years [43]. Unexpectedly, at the peak of the immune response, it was elucidated that more than 90% of the CAR T cells proliferated from a single clone with the disruption of the methylcytosine dioxygenase TET2 gene because of a lentiviral vector-mediated CAR transgene integration [43]. TET2 knockdown corroborated its direct negative effect on the differentiation of CAR^+^ CD8^+^ and CAR^+^ CD4^+^ primary T cells, which subsequently illustrated the TET2 gene as an epigenetic regulator of T lymphocytes [43]. TET2 mutations have been linked to the FOXP3 expression level reduction and instability in T cells, resulting in a decrease in the population of T regulatory (Treg) cells and an increase in the population of T-helper (Th) 1 cells and Th17 cells. This shift in T-cell polarization leads to a greater antitumor activity as well as a higher risk of autoimmune disorders [43]. These findings propose that the progeny of a single CAR T cell may lead to leukemia CR alongside introducing TET2 as a potential target gene of site-specific integration for improving CAR T cell immunotherapies [43, 44]. In addition to TET2, there are several other genes that CAR transgene integration into them might render CAR T or CAR NK cells more efficient and persistent in the recipient, leading to higher remission rates. The candidate genes are presented in Table 1.

**Table 1:** Specific gene editing can improve the characteristics of CAR T/NK cells

Gene(s)	method	Cell type	T/NK cell Improvement	Reference
TET2	Disruption by CAR transgene	T cells	TET2 dysfunction results in the production of effective CAR T cells, which have the characteristics of short-lived memory cells that can mediate effector responses, as well as long-lived, persistent memory cells	[43]
UBR1	Disruption by CAR transgene	T cells	A member of the ubiquitin ligase family is involved in protein degradation and contributes to the formation of long-term persisting clones	[45]
STAT5B and BACH2	HIV-1 insertional activation	T regulatory and T central memory cells	Genes commonly targeted as insertion sites by HIV-1, generate chimeric mRNAs that are enriched in T regulatory and T central memory cells, and increase proliferation and survival rate without compromising function	[46, 47]
TRAC (CD52, dCK)	Disruption by CAR transgene or by TALEN	T cells	Reduces tonic signaling, avoids an accelerated T cell differentiation and exhaustion, improves the therapeutic efficacy, renders T cells resistant to simultaneous infusions of lymphodepleting regimens, and controls the rate of elimination via host versus graft reactions	[25, 48, 49]
PD-1 (B2M, TRBC, TIM-3, LAG-3, A2AR)	Disruption by multiplex genome editions	T cells	Generate universal CAR T cells resistant to PD-1 inhibition and improves antitumor efficacy	[50–56]
TOX/TOX2	Disruption /CAR-T cells generated from donor TOX and TOX2 DKO (-/-) mice	CD8^+^ T cells	CAR TILs deficient in both TOX and TOX2 are more effective than wild-type (WT) in suppressing tumor growth and prolonging the survival of tumor-bearing mice	[57]
NR4A	Disruption	CD8^+^ T cells	CAR T cells that are lacking all three Nr4a TFs (Nr4aTKO) promote tumor regression and prolong the survival rate of tumor-bearing mice and reduce hyporesponsiveness of CD8^+^ T cells	[58]
P38	Disruption /using p38i in culture media	T cells	Pharmacological inhibition of p38 improved the efficacy of mouse anti-tumor T cells and enhanced the functionality of human tumor-reactive and gene-engineered T cells	[59]
HPK1	Disruption by CRISPR/Cas9	T cells	less exhausted and more active and proliferative T cells	[60]
IFN-γ signaling genes	Disruption	NK cells	Known to improve NK cell function	[61]
CD5	Disruption by CRISPR/Cas9	Jurkat cells	CAR T cells deficient in the expression of CD5 do not mediate fratricide	[62]
shp-2	Disruption by CRISPR/Cas9	NK-like YT cells	Increases the cytotoxicity of effector NK-like YT cells	[63]
TGFBR2 (FOXP3)	Disruption by CRISPR/Cas9	NK cells T cells	Modified NK cells become TGFβ1-resistant, exhibit increased proliferation and effector cytokine production, long-term persistence, as well as increased ability to mediate eradication of aggressive tumor	[64–66]
HPRT1	Disruption by CRISPR/Cas9	Primary NK cells	Modified NK cells become resistant to TGFβ1	[64]
ADAM17 and PDCD1	Disruption by CRISPR/Cas9	Primary NK cells	Significantly improves activity, cytokine production, and tumor cell cytotoxicity	[41]
CISH	Disruption by CRISPR-Cas9	Cord blood NK cells	Targeting a cytokine checkpoint further enhances the antitumor activity of IL-15-secreting armored CAR-NK cells by promoting their metabolic fitness and antitumor activity	[67]
TIGIT	Blockade	NK cells	TIGIT inhibits NK cell cytotoxicity by opposing CD226, so its blockade can lead to persistent therapeutic benefits	[68, 69]
SHP-1	Blockade	T cells	Better control of PD-L1 expressing tumor growth alongside increasing the infiltration rate of CAR T cells into the tumor milieu	[70, 71]
A2ARs	A2AR antagonists or targeting of A2AR using shRNA	T cells	Inhibits T cell activation through the cAMP-PKAI-CSK pathway; therefore, its inhibition enhances anti-tumor effects mediated by CAR T cells	[72]
ROS family	Disruption	T cells	Causes DNA oxidative damage	[73]
HDACi	Augmentation	T cells and NK cells	Can lead to survival potency in CAR T cells along with immunoradiotherapy	[74, 75]
p53	p53-KO T cells from donor transgenic mice	T cells	As a tumor suppressor protein cause upregulation of PD-1 and its PD-L1 and with redox activity may enhance T cell radioresistance	[76]
BCL2 family	Disruption	T cells	Prevents the intrinsic apoptosis and synergistically enhances the persistence of T cells, reduces their sensitivity to Fas-induced apoptosis, alongside increasing their survival and antitumor activity	[77, 78]
SMAD3	Knocking down	NK cells	Its silencing improves NK cell cytotoxicity in solid tumors	[79]
CCR5	Disruption by ZNF	T cells	A safe harbor locus	[80]
CD56	Augmentation	T cells	The homophilic interaction between intercellular CD56 correlates with enhanced infiltration of CAR T cells, increased secretion of INF-γ, and prolonged survival of CAR T cells. Moreover, ectopically expressed CD56 promotes CAR T cell survival and antitumor responses	[81]
CD73	Blockade	NK cells	Increases homing of NKG2D-CAR NK cells to tumor sites and improves antitumor responses in animal models	[82]

Eyquem et al. demonstrated that integrating the CAR-encoding sequence into the TCR gene and under the control of its endogenous regulatory elements control the transcriptional regulation of CAR expression in the same manner as that of the endogenous TCR, which is crucial for desirable T cell function and tumor eradication [25]. Also, Stenger et al. observed that the loss of the CD8^+^ CAR T cell efficacy is related to T cell exhaustion and apoptosis, while TCR antigens were still present in CAR T cells [25, 83]. The gene expression profile confirmed that CD8^+^ CAR T cells become more exhausted and apoptotic following a CAR engagement with the target antigen and the TCR gene expression stimulation [25]. Therefore, the *TCR* locus can be considered one of the most interesting gene targets for the integration of the CAR transgene [25]. In conclusion, the disruption or enhancement of the expression of some specific genes could be effective in improving the efficacy of CART/NK cells, such as increasing antitumor functionality, in vivo persistence, and efficient proliferation capacity, alongside postponing T cell exhaustion [50, 84]. Generally, to achieve all these goals, we should be familiar with various site-specific integration technologies, which will be discussed in the upcoming section.

## Site-specific integration methods

**Fig. 2:**
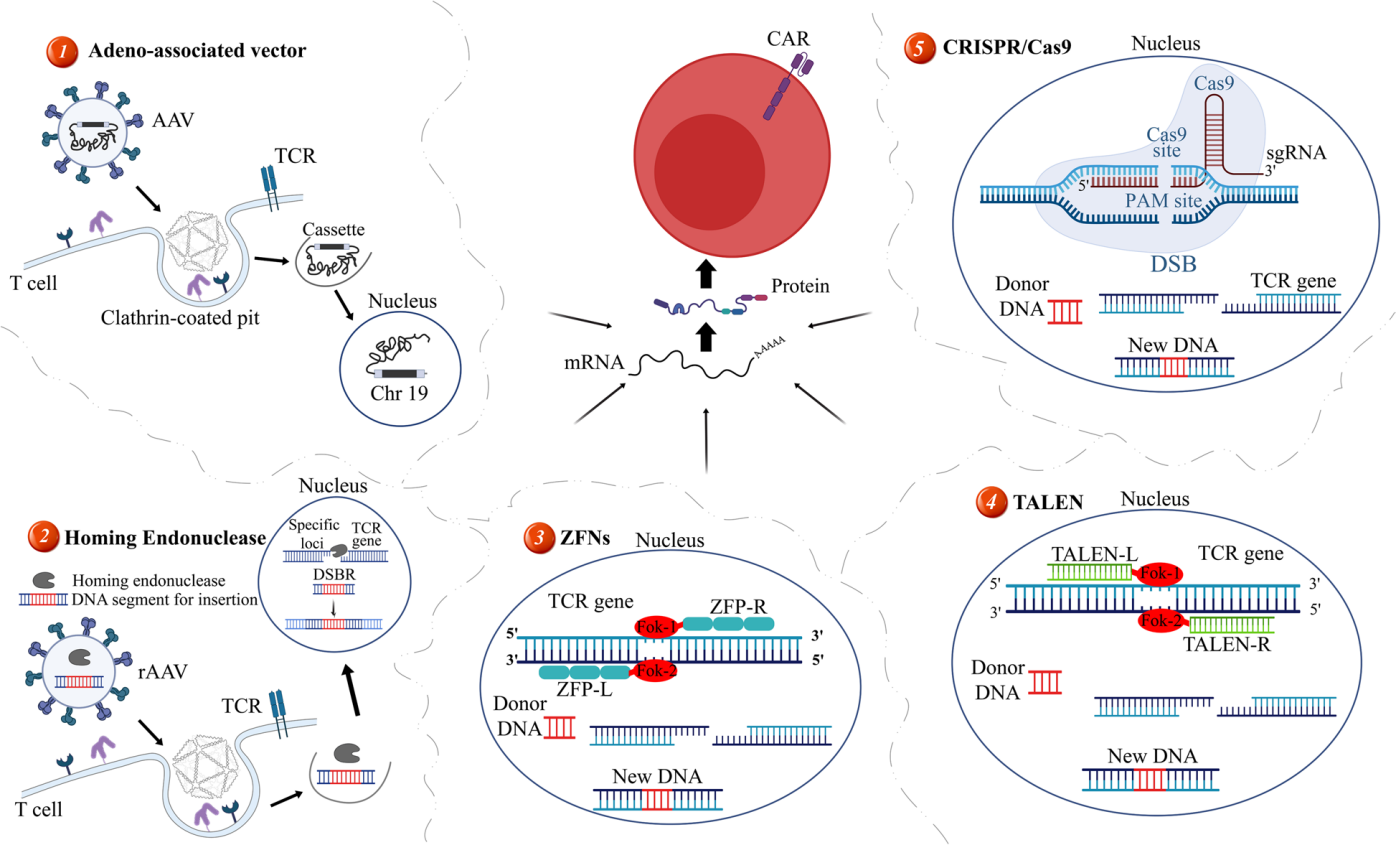
Different methods for the generation of CAR T cells with site-specific CAR transgene integration. Defined locations are targeted in the T cell genome that are considered safe and efficient harbors for CAR transgene integration using different methods as follows: 1: Adeno-associated vectors mediate the delivery of CAR DNAs into T cells as well as their integration into the *AAV1* loci on chromosome 19 through binding their receptors on the surface of T cells. 2: Recombinant Adeno-associated viruses (rAAV) accompanied by engineered homing nucleases can edit the T cell genome at sites that are specified by engineering a homing nuclease that transports and places a CAR construct precisely at the desired location. 3, 4, and 5: The latest genome-editing technologies that can be used to insert CAR transgenes into desired target locations in the T cell genome

Several efforts have been made to improve the efficiency of the CAR T/NK cell therapy, such as gene-editing techniques [85]. Recent advances in genome-editing technologies enable targeted integration of any desired gene fragment with various different functions [85]. Gene-editing technologies depend on the employment of engineered nucleases to make DSB in defined target DNA sequences [15]. DSB is repaired by endogenous cellular enzymes in two ways: one is non-homologous end joining (NHEJ), which is an error-prone pathway resulting in a high frequency of nucleotide insertions or deletions (indels), and the other is homologous direct repair (HDR) in which the DSB is repaired by a homologous DNA strand as a template [15]. The HDR can be reversed by exploiting an exogenous donor DNA fragment as a template to insert into a specific DSB site, which results in the integration of the desired non-homologous sequence flanked by homologous sequences [15]. Although, gene editing can be applied to disrupt a gene function by indels generation, an HDR is required to insert new coding sequences [15]. However, in the case of CAR T cell therapy, both goals are desired. The HDR process precisely enables a targeted CAR DNA replacement at the designated target site [15]. Targeted nucleases, which include homing endonuclease, ZFN, TALEN, and the CRISPR technology, are the most common and powerful classes of enzymes that enable genome editing through the creation of a site-specific DNA DSB at a pre-defined site in the genome [15]. Here, we summarize the systems used to produce CAR T cells with a site-specific integrated CAR transgene through genome-editing platforms as well as efforts to pave the way for this goal (Fig. 2) [15].

### AAV vectors for transferring CAR transgenes

Wild type AAVs are able to preferentially integrate their genome into the human chromosome 19 (19q13.42) at a site that is referred to as the AAV Site 1 (AAVS1) locus [86]. Their genome contains the genes of *Rep* (required for DNA replication) and *Cap* (required for capsid formation), both flank by inverted terminal repeats (ITR); therefore, once inside the cell, the genetic material primarily remains in an episomal conformation [87]. The AAVS1 locus overlaps with the first exon of the PPP1R12C gene (which encodes the protein phosphatase 1 regulatory protein subunit 12C) [86].

Zhang et al. created a non-viral vector termed "*CELiD*" DNA (a closed linear duplex) from the AAV genome [88]. This vector bears the CAR expression cassette which is flanked by the AAV ITRs. To achieve safe and long-term CAR expression, the specific integration of the CAR transgene into the AAVS1 site introduced by the CELiD vector was studied. CELiD DNA was produced from Sf9 cells under the rescue and replication of ITR-flanking open reading cassettes mediated by AAV-Rep protein [88]. Unlike encapsidated AAV vector genomes, CELiD DNA has no packaging constraints, which limit the space within the viral capsid [88]. Genetic analysis revealed that the insertion site of the CAR transgenes was preferentially located in the AAVS1 in Jurkat cells as well as primary T cells [88]. In vitro analysis of the CAR expression and secreted cytokine levels from the engineered T cells showed abundant amounts of cytokines secreted in comparison with control T cells [88]. CD19-redirected CAR T cells were fully functional and they mediated cytokine secretion and killing of CD19^+^ cells as high as 75% in vitro [88]. rAAVs containing a gene of interest can be produced, independently of adenoviral co-infection, by expressing Rep, Cap, and adenoviral helper genes in trans while inserting the gene of interest between the ITRs [89]. Investigations revealed that recombinant vectors, bearing either the AAV ITRs or the AAV2-derived p5IEE, have a good potential to site-specifically integrate at the AAVS1 locus [89]. Overall, a site-directed AAV vector might be efficiently used in the human T cell engineering, and it might enhance the safety index of CAR T cell therapy.

In the genome editing of T cells, the donor template can be a single-stranded DNA, a double-stranded DNA, or a short oligonucleotide [90]. Given that AAV-based vectors can efficiently package genomes up to 4.7 kbp, this makes AAV vectors very suitable for transgene integration in CAR-based platforms [90]. Intriguingly, the template DNA fragment for a T cell engineering must use the HDR mechanism, which is mostly active during the cell cycle phases S and G2. In this regard, AAV transduces cells in the S phase [90]. Among the broad range of AAV capsid serotypes, Wang et al. suggested both CD8^+^ T cell and CD4^+^ T cell subsets are highly permissive to AAV serotype 6 (AAV6) transductions [90]. When they attempted to prepare a CAR expression cassette using plasmid DNA or PCR products as the template, the efficiency of gene integration was less than 10-fold in comparison with an AAV [91]. They supposed that might be due to the AAV capability to obtain higher intracellular concentrations, or the interaction of the virus with host factors involved in HDR [88]. There are some FDA approved drugs that are based on AAV vectors, including *Zolgensma*, *Luxturna*, *Gendicine*, and *Oncorine* [92]. Moreover, MacLeod and colleagues demonstrated that using engineered homing endonucleases and AAV HDR templates can be beneficial in the production of effective allogeneic CAR T cells by inserting a CAR transgene into the exogenous TCR locus in an efficient and simplified process [51]. The process was carried out to generate CD19-redirected CAR T cells that have a strong tumoricidal activity in a disseminated lymphoma model, eliminating tumor cells in the moderate and high-dose cohorts [51].

### Gene targeting methods

#### Homing endonuclease

The homing endonuclease is a highly specific double-stranded DNase with a large asymmetric recognition site from 12 to 40 nucleotide lengths, and its coding sequence is regularly placed within introns [93]. Unlike the common restriction endonucleases, homing endonucleases can recognize degenerative sequences in a way that a single nucleotide change could not prevent them from cleavage but might diminish their ability to some extent [94]. The use of homing endonucleases is not as popular as other gene-editing tools (such as ZFN, TALEN, and CRISPR/Cas9) because they are difficult to engineer. Despite these challenges, MacLeod and colleagues studied homing endonucleases [51]. They observed some structural and mechanistic advantages that make them interesting for in vivo and ex vivo genome editing [51]. They can be generated by a small single peptide, 310 amino acids, an enzyme called TRC1-2, which makes it easy for the nuclease to pass into cells [51]. In addition, what is particularly relevant to this study is that the engineered homing endonuclease cuts the DNA at the TRAC locus, leaving 4-bp 3' overhangs on the two strands at the DSB site [51]. It has been noted that the 3' overhang contributes to the HDR, which might partly explain the high CAR insertion rate observed in this study [51].

#### ZFN

ZFN is a type of DNA-binding protein that has been engineered to mediate genome editing by generating DSBs at specified locations [95]. ZNFs are capable multimers in a way that each separate finger recognizes three to four base pairs in the DNA sequence of the genome. The cooperation of several zinc fingers can create highly specific recognition sites [95].

Brown et al. produced an IL-13Rα2-specific CAR, named *IL13-zetakine* [96]. This CAR distinguishes mutated IL-13Rα2 at a single site (E13Y) to diminish the possibility of binding to the other commonly expressed IL-13Rα2 structures [96]. Using a ZFN to knock out the glucocorticoid receptor gene in the  CAR^+^ cytolytic T cells (CTLs), they inhibited the apoptosis of IL-13Rα2-specific CTLs in patients with glioblastoma on steroids. They believed that ZFN-modified glucocorticoid-resistant IL-13Rα2-specific CTLs could maintain function in patients treated by glucocorticoids [96]. In addition, when administered in the presence of glucocorticoids, these CAR T cells could be prepared from allogeneic sources for the possible treatment of recurrent glioblastoma multiform (GBM); however, more in-depth preclinical and clinical investigations must be conducted in this regard [96].

#### TALEN

TALEN is a natural protein of the Xanthomonas, a pathogenic plant bacterium [97]. It contains a DNA binding domain composed of 33–35 amino acid repeating domains, and each domain can recognize a single nucleotide [98]. The specificity of TALEN is determined by two hypervariable amino acids, which are called repeated variable di-residue (RVD) [98]. Similar to ZNFs, modular TALEN repeats are joined together to recognize consecutive DNA sequences [98]. Nevertheless, different from ZNFs, there is no need for re-engineering the flanked repeated sequences with the capacity to recognize specific sites in the genome [98].

Sather et al. designed a hybrid nuclease that combined a DNA binding domain TALEN with an engineered homing endonuclease, highly sequence-specific (named *megaTAL*) [99]. They used a megaTAL nucleases and a AAV6 donor vector to develop highly effective CAR T cells by HDR at two loci including HIV co-receptor chemokine (C–C motif) receptor 5 (CCR5) and TCRα [99]. CAR T cells produced by this method might be applicable in HIV^+^ patients with lymphoma, where a simultaneous CCR5 disruption could protect the therapeutic cells from the HIV infection, as well as an off-the-shelf therapy [99].

Poirot et al. reported that TALEN-engineered TCR^Knockout^ CAR T cells do not mediate GVHD, and that elimination of the TCR would not negatively influence the anti-tumor function of CD19-redirected CAR T cells [48]. In another study, Valton and colleagues described universal TALEN-engineered CAR T cells (UCART19) which were then used in two children with B-cell acute lymphoblastic leukemia (B-ALL) [49]. In detail, there was no sign of GVHD, and the patients were in CR via molecular evaluations 12 months following the treatment [49]. Furthermore, Poirot and colleagues demonstrated that disrupting the expression of CD52 (the target protein of *Alemtuzumab*) in CAR T cells by TALEN can result in the development of alemtuzumab-resistant CAR T cells [48]. In addition, since CD52^Knockout^ CAR T cells are resistant to depletion by alemtuzumab, it would enable CD52^Knockout^ CAR T cells to be used after or in combination with *alemtuzumab* to target host cells and enhance an adoptive T cell therapy [100]. In a clinical trial report by Qasim and colleagues (NCT02808442), two R/R B-ALL children received a single-dose treatment of UCART19 cells followed by lymphodepleting chemotherapy (fludarabine 90 mg/m, cyclophosphamide 1.5 g/m and alemtuzumab 1 mg/kg) and serotherapy with anti-CD52 [101]. In this trial, both of the patients achieved molecular remission after 28 days [101].

#### CRISPR/Cas9

In recent years, the discovery of the Cas9 nuclease guided by a short RNA sequence opened a new window to the genome-editing technology [102]. The CRISPR/Cas9 system can be applied to mediate an effective eukaryotic genome engineering through the simple design of a 20-nucleotide within its guide RNA specified for the pre-defined target sequence [102]. CRISPR/Cas9 employs the HDR machinery in mammalian cells to minimize off-target cleavage alongside offering more specificity [102].

Recently, an effective homologous recombination by AAVs and a CRISPR/Cas9 system was shown to mediate the site-specific integration of large CAR transgenes into the T cell genome [25]. In detail, a CD19-redirected CAR transgene was successfully integrated into the TRAC locus as an attempt to disrupt the TRAC gene and place the CAR transgene under its transcriptional control [25]. For this aim, Eyquem and colleagues employed a gRNA specific for the 5' end of the first exon of the TRAC gene, alongside an AAV vector [25]. They electroporated the Cas9 mRNA into the T cells [25]. This well-organized targeting method was first reported by Eyquem et al. in which insertion was at the TCR locus, and is comparable to the engineered CAR T cells with the CAR transgene insertion at the AAVS1, CCR5, or CD40L positions [25]. Moreover, approximately, 95% of the CAR^+^ cells were deficient in the expression of TCRs [25]. However, in vitro functional studies did not find any significant difference between randomly integrated 19-28z CAR T cells and those whose CAR transgenes were integrated into the TRAC locus [25]. Moreover, CAR T cells under the regulation of the TRAC promoter mediated better antitumor responses and prolonged median survival in comparison with the randomly integrated CAR group (integration via retroviral vectors) in preclinical mouse models [25].

According to another study, Baylor College of Medicine’s research team devised a method to develop CAR T cells to target T cell antigens [103]. The main obstacle to designing T cell immunotherapy against T cell-based oncological indications is that these engineered T cells are susceptible to fratricide (comprehensively discussed elsewhere) [104]. They employed CRISPR/Cas9 to knock out the T cell-specific antigen CD7, and then to engineer the CD7-deficient T cells to express CARs redirected against CD7 [103]. These CD7-redirected CAR T cells showed an efficient functionality in vitro which corroborates the feasibility of this platform [103]. However, the immunodeficiency caused by the pan-T cell depletion seems as an important obstacle for the clinical translation of this strategy [103].

It is necessary to mention only a few CRISPR/Cas9 studies have been published in regards to NK cells [64], in one of which, TGF-βRII expression was abrogated at both, mRNA and protein levels [105], demonstrating improved CAR NK cells persistence and resistance to the TME.

Multiple genome-editing technologies can effectively generate clinical-scale gene-disrupted CAR T cells with effective antitumor activity and reduced alloreactivity, which might be used as off-the-self universal T cell therapeutics [106]. A precise genome editing might be important when the cellular target is a long-lived cell such as memory T cells in which CAR integration into a specific locus could provide a safer and more distinct T cell product, as well as more potent CAR T cells by enabling a constant CAR expression and preventing batch-to-batch variations [106]. This strategy could also lead to the prevention of vector copy number variation, minimize the risks of insertional oncogenesis, gene-induced autoimmunity, and alloreactivity, and reduce constitutive signaling, alongside delaying T cell exhaustion [106]. Despite of all these advantages, each method also has its disadvantages, which we will review in the following section.

## Disadvantages and counterstrategies

Even though the CRISPR technology has solved many of the limitations of the conventional CAR T cells, safety issues must be resolved before these gene-edited cells enter the clinic [107]. Various factors such as off-target effects, nuclease activity, target site preference, gRNA design, and delivery methods can determine the efficacy and safety of such genome-editing systems [108].

The first consideration in gene-editing methods is the issue of “off-target”. This off-target incidence might be beneficial for bacteria and archaea [109]. However, some recent studies have demonstrated large genome deletions or inversions induced by unintentional gene editing in several animals, including mice, C. elegans, and rabbits [110]. To approve every clinical treatment method for humans, clinical safety is the most important issue for every regulatory agency. Some recent studies have reported off-target effects of gene editing in T cells [111]. Off-target effects introduce random mutations; thereby affecting tumor suppressor genes or activating oncogenes [112]. According to a report, when CRISPR/Cas9 was used to insert a transgene into the TRAC or TRBC locus of CAR T cells, off-target effects were also observed [113]. Another study has shown that when the whole genome of CRISPR/Cas9-edited mouse models was sequenced, it was elucidated that gene editing could lead to hundreds of unexpected mutations in the genome [114]. It is also worth noting that another study showed genome editing can cause DNA damage through the p53 protein in human retinal epithelial cells [115]. The activation of p53 protein may lead to chromosomal rearrangements and other tumorigenic mutations in cells [115]. Even though the result of the p53 protein activation induced by genome editing is uncertain, it seems that it contributes to reducing the efficiency of gene editing [115]. Therefore, off-target issues must be considered in the future development of genome-edited CAR T cells [115]. Off-target analysis should be performed during the target selection process of gene editing to manage the safety risks associated with development of gene-edited CAR T cells [116].

Theoretically, the idea of multiplexed CRISPR systems for various biological engineering purposes seems interesting; however, numerous issues question the real-life practicality of such systems. One of the most prominent struggles in this regard is predicting the behavior of multiple gRNAs simultaneously present in the context of a functional cell. Recently, researchers have come up with solutions to solve such problems. For instance, Reis and colleagues devised an array (named extra-long sgRNA arrays; ELSAs) that enabled 22 sgRNA coexpression for the repression of a maximum of 13 genes [117]. These researchers were also able to engineer the phenotype of *Escherichia coli* in three ways; abrogating amino acid synthesis, manipulating metabolism to elevate succinic acid synthesis, and quenching responses to stress conditions [117]. Another issue associated with the application of multiplexed CRISPR systems is that multiple cleavages culminate in unfavorable chromosomal rearrangements. Some researchers attempted to address this issue by designing different gRNAs that mediated distinct chromosomal cleavages and by in-depth analysis demonstrated that such occurrences could be anticipated beforehand [118]. As the application of CRISPR systems increased in biological systems, researchers introduced an occurrence termed retroactivity [119]. Retroactivity is defined as when gRNAs in a given cell are numerically increased, their competition for nucleases also escalates, an effect that consequently lowers the efficacy of the given gRNAs [119]. For a CRISPR-Cas9 system to work, a complex needs to be formed between the gRNA and the endonuclease so that they can detect the target genetic site and exert their mission. Due to the ultra-sensitivity of such complexes (which are as specific as the length of 5 nucleotides with the spacer), their application can result in serious off-target effects, especially as gRNAs are increased in number [120, 121]. Researchers have developed various counterstrategies to resolve this issue, which include designing of gRNAs with shorter spacers [120, 121]. Ran and colleagues also engineered a mutant form Cas9 that functions as a nickase with a pair of gRNAs as they mediate DSBs leading to highly specific cleavages in the genome [122]. These researchers demonstrated that this strategy could minimize off-target cleavage effects in cell lines by up to 1500-fold [122]. According to another study, Guilinger and collaborators reported the development of a fusion protein of Cas9 and FokI (named fCas9) that requires two fCas9 monomers to separately bind their specific genetic sites at the same time for a targeted cleavage [123]. The researchers demonstrated that the specificity of fCas9 is more than 140-fold greater that the wild-type version of Cas9 [123]. Anzalone and colleagues stepped even further by designing a prime editing platform in which a catalytically null form of Cas9 was engineered with a reverse transcriptase and a gRNA for direct genetic manipulation and reported that using this system, they were able to carry out site-specific integration/deletion alongside all types of points mutation, obviating the need for DSBs or genetic templates [124]. Despite all these advances in this field, researchers need to focus more deeply on assessing the applicability of these strategies and platforms in vivo, alongside devising counterstrategies for the issues that arise thereafter.

Because of such off-target effects, the safety of engineered CAR T cells is the primary concern. To minimize the mentioned safety risks, the target site needs to be carefully selected [125]. However, there is sufficient literature to prove that DSBs are serious damages that can drive genome instability and cell death [126]. In the case of multiple gene editing, the concerns surrounding multiple DSBs are further exacerbated, where multiple DSBs that exist simultaneously may increase toxicity [108]. The association between the number of disconnected DSB sites and the results of potential translocations could highlight this point. Although such events are rare in T cells, necessary analysis should be carried out to ensure the safety of gene-edited CAR T cell products. In addition to the safety risks of translocation, functional changes of gene-edited CAR T cells are most likely to cause adverse events in patients [127].

Alongside participating in the HDR pathways, AAV can also insert genetic materials at the site of a DSB via the NHEJ mechanism to reach accurate on-target genome editing [128]. When it occurs at the expected target site, such an event can be considered as an on-target gene addition; however, when it occurs at a DSB generated by a random cellular event or off-target nuclease activity, such events are considered off-target events [129]. HDR is the main repair pathway for T cell genome engineering which can be combined with nuclease mRNA and AAV6 donors [91, 130].

To achieve an HDR-mediated gene editing that may have therapeutic benefits, several steps need to be optimized. First, a well-tolerated method must be developed to introduce targeted nucleases into the desired cells to produce sufficiently high levels of DSBs. Of note, the delivery of ZFN as mRNA by electroporation appears to be very effective with minimal cytotoxicity [131]. Additionally, a homologous donor template has to be chosen. The ideal donor should be selected by designing and testing several donor variants with different DNA homolog arms to confirm that the selected construct can provide an effective HDR-mediated genome editing. If the target site used for gene correction or transgene addition is distant from the nuclease cleavage site, the size and position of the homology arms are particularly important, because this may lead to a greatly reduced level of HDR-mediated genome editing [132].

Several in vitro and in vivo studies have shown that TCR^-^/CAR^+^ T cells are highly functional with no alloreactivity in comparison with CAR T cells that have endogenous TCRs [25, 83]. In fact, CAR T cells with or without TCRs enhanced survival rates in a xenograft mouse model but CAR T cells showed prolonged and sustained persistence in vivo only while proficient in the expression of endogenous TCRs; therefore, the endogenous TCR might play a role in the activation or stabilization of T cells [83]. This creates a dilemma for researchers as they would have to decide between the prolonged persistence of the engineered CAR T cells and host alloreactivity responses (Table 2).

**Table 2:** Site-specific integration in CAR-T/NK cell therapy

Advantages	Disadvantages
A better defined T cell product Safer therapeutic T cells Functional improvement of CAR T cell therapy Enhancement of some desirable genes Avoiding position-effect variegation Controlled integration of the foreign DNA in the genome	Target site selection and sgRNA design Cas9 activity Delivery methods Simultaneous DSBs can lead to cytotoxicity Gene disruption in CAR T cells can cause unintended innate immune responses DSBs are toxic and can drive genomic instability and cell death
Continuous CAR expression (random integration into the genome causes substantial variations in CAR expression levels in a batch of CAR T cells because of different transgene copy numbers per cell)	**Unpredicted translocations** (may occur between double-strand breaks when multiple genes are edited)
Disrupting tumor microenvironment (TME)-driving immunosuppressors Knocking out of genes targeted by immunosuppressive drugs Knocking out of genes targeted by radiotheapeutic or chemotherapeutic agents Knocking out of genes responsible for T cell apoptosis to enhance cell survival	**Off-target effects** (introduction of random mutations, thus impacting tumor-suppressor genes or activating oncogenes)
Minimizing the risks of insertional oncogenesis	
Allowing allogeneic CAR T therapies (due to the disruption of genes involved in Graft-versus-Host Disease)	
Effective against solid tumors (due to the disruption of genes involved in immunosuppressive pathways)	
Reversing T cell exhaustion	
Ablating Fas-induced cell death (using site specific gene-editing methods might lead to an enhancement of CAR T cell function)	

Another safety concern is associated with the innate immune responses in host cells triggered by the gRNAs of CRISPR systems. According to report by Kim and collaborators, it was elucidated that CRISPR gRNAs harboring a 5'-ppp group and developed by in vitro transcription contribute to the formation of immune responses in human cells as they are sensed by a protein called DDX58, an occurrence that culminates in the orchestration of type I interferon responses leading to the mortality of a high percentage of cells [133]. Moreover, it was demonstrated that the mentioned phosphate group can be eliminated by an enzyme in vitro, and that the produced gRNAs could still be functional (while coupled with Cas9) in terms of producing targeted genetic mutations at a high rate in human T cells proficient in the expression of CD4 without mediating immune responses [133]. Such immune responses might occur in a population of T cells destined for CAR T cell manufacturing, and researchers need to more precisely focus on this matter and elucidate the aspects of such unfavorable events in the context of CAR T cell development.

## Appropriate safe harbors for CAR transgene integration

The reliability and safety of a gene integration for therapeutic cell engineering purposes will be limited due to the interaction between the genome of the target cells and the exogenous genetic materials [16]. Aside from the fact that the delivery of the target genes has made great progress in CAR T cell therapy, there is still insufficient knowledge as to how to integrate foreign DNA fragments into the genomic DNA to have the highest rate of safety and effectiveness [16]. In this section, we will discuss appropriate GSHs where CAR transgenes can be integrated and then they would operate in a predictable manner without interfering with the endogenous gene activity or mediating oncogenic chromosomal translocations.

Extensive research on gene insertion provides details of insecure integration harbors [134]. How do we determine the regions of the genome that should be avoided during transgene integration in gene therapies? To perform safe genetic correction, which genes should be avoided due to being cancer-related genes? Should a GSH be located in the selected gene or not? The definition of GSH here reflects the lessons learned from the side effects associated with insertional mutagenesis, as well as proper CAR expression and ideal T/NK cell phenotypes [135]. The important issue for CAR-based therapeutics is to achieve desirable T/NK cell phenotypes with the highest efficacy and persistence. In the upcoming section, we discuss some putative criteria to select GSHs to generate CAR T cells [136].

### Principle criteria for GSHs

Over the past decades, numerous research teams from around the world have dedicated a great deal of effort to set criteria for GSHs. For instance, Papapetrou and colleagues conducted research on GSHs by focusing on the transgenic expression of β-globin in induced pluripotent stem cells (iPSCs) [137]. Upon lentiviral transduction, the researchers reported that around 10% of the integrated transgenes were in GSH which enabled their elevated expression without interference with the expression of surrounding genes [137]. Papapetrou et al. based their definition of GSH on five characteristics by using in silico and in-depth analysis which could be useful for other researchers in the context of genetically modified cell-based therapies [137]. Since transactivation of pro-tumor genes is the most frequent occurrence in the context of insertional oncogenesis, the first two GSH criteria proposed by Papapetrou et al. would take this matter into consideration by suggesting a distance of ≥ 50 kb from any given gene (1^st^ criterion) or ≥ 300 kb from any cancer-associated gene (2^nd^ criterion) [137]. Moreover, since it has been demonstrated that miRNAs play critical roles in the maintenance of various cellular and molecular mechanisms, the researchers based the 3^rd^ criterion on the distance of ≥ 300 kb from any miRNA genes [137–139]. Papapetrou et al. introduced the 4^th^ exclusion criterion as that any integration should not be within a transcript unit, based on the fact that transgene integration into transcription units might contribute to tumor suppressor loss of function or the emergence of abnormal gene products due to abnormal splicing [137]. Ultimately, the researchers suggested the ultraconserved regions of the genome must not be considered for transgene integration since they might be rich in various functional genetic elements (5^th^ criterion) [137].

Recently, Odak and colleagues conducted a study to assess an algorithm for identifying extragenic GSHs (eGSHs) in human T lymphocytes that could be leveraged for CAR transgene integration to achieve sustainable CAR expression avoiding spontaneous CAR stimulation and T cell terminal differentiation [140]. The researchers based their algorithm on seven criteria as an attempt to diminish transgene integration-related cytotoxicity by avoiding integration into operative genomic elements, preventing transgene silencing, and increasing the efficacy of CRISPR-Cas9 [140]. Because of the pronounced importance of non-coding RNAs (ncRNAs) in various cellular functions, a 6^th^ criterion was introduced so that transgene integrations should not result in the perturbance of ncRNAs [140–142]. It has been demonstrated that some ncRNAs play vital roles in various cellular and physiological processes, including gene expression and regulation, chromatin dynamics, differentiation, and development [143]. The disruption or dysregulation of ncRNA may cause cancer and immunological disorders. For example, HOTAIR encodes a long non-coding RNA (lncRNA) that regulates key epigenetic regulators and silencing, and its dysregulation may lead to cancer formation and other issues [144].

To achieve an efficient site-directed transgene introduction into the genome, the respective nuclease must have effectual accessibility and cleavability to the targeted site (7^th^ criterion) [140]. Ultimately, Odak and colleagues also introduced an 8^th^ criterion based on chromatin structure in a way that it does not interfere or suppress the desired expression and regulation of the introduced transgene [140]. Furthermore, Odak and colleagues reported that T cells engineered for the expression of CD19-redirected CARs (at a GSH called *GSH6*), for the transgene integration of which all of the suggested criteria were taken into consideration, were effective in preclinical mouse models of B-ALL at low doses, alongside being capable of resisting tumor rechallenge 100 days following their administration [140]. Moreover, these CAR T cells were reported to be comparable in terms of efficacy to CD19-redirected CAR T cells whose CAR transgene were integrated into the TRAC locus [140]. Such investigations and findings further accentuate the importance of GSHs for gene engineering-based cell therapies, and that future studies could be conducted in this direction for having more effective and less toxic therapeutic interventions [140].

### Some specific GSH examples

Although, there is not a perfect matched site, there are some reports in reference to integration sites and the effects of transgene expression on the neighboring genes. So far, three sites were introduced as the target site of CAR integration as GSHs: (1) AAVS1; (2) the CCR5 gene; (3) the human Rosa26 locus.

#### AAVS1

The AAVS1 locus (position 19q13.42, in the human genome) is a common integration site of the AAV, which has been identified as a nonpathogenic safe-harbor location for robust transgene expression [145]. The widespread expression across cell types may be attributed to the DNase I hypersensitivity sites and insulator elements in the AAVS1 locus, which can maintain an open chromatin conformation [146]. Importantly, the AAVS1 locus is a gene-rich region and some integrated promoters can indeed activate the neighboring genes [147]; however, their exact function in different tissues is currently unknown [148]. On the other hand, this indicates that the transgene integrated into the AAVS1 region shows strong expression which remains stable in CAR NK cells derived from iPSC [149]. Moreover, CAR insertion into the AAVS1 site disrupts the phosphatase 1 regulatory subunit 12C (PPP1R12C) gene and the consequences of its haploinsufficiency or deactivation in some cells have been investigated [150]. So far, it has been elucidated that AAVS1 is a special site where the integrated CAR transgene can be stably expressed without pathogenicity in engineered human T/NK cells [151].

#### CCR5

CCR5, also known as CD195, is a protein on the white blood cell surface that is involved in the immune system and acts as a receptor for chemokines [152], and the major HIV-1 co-receptor [153]. The discovery that homozygosity for a naturally occurring null mutation (CCR5Δ32) confers resistance to an HIV-1 infection indicates biallelic disruption of CCR5, which is desirable for an effective HIV resistance and should not be detrimental to T-cell function [154]. This feature potentially makes the CCR5 locus a favorable target site for other genetic engineering-based T-cell therapies, because this site does not affect cell survival or growth, and is located within open and transcriptionally active chromatin [155]. HIV^+^ patients are at increased risk for B-cell lymphomas and plasma cell disorders [156]. These malignant tumors can be targeted using CD19-redirected or BCMA-redirected CAR T cells, but in these patients, T cells are susceptible to HIV infection [157]. However, CAR T cells deficient in the expression of CCR5, due to the CCR5 gene disruption, might be the solution. In this regard, CCR5-disrupted CAR T cells have been investigated, and more in-depth studies are warranted in this regard [158, 159]. Due to the inherent susceptibility of T cells to HIV infection, HIV^+^ patients are excluded from ongoing CAR T cell clinical trials [156]. Like AAVS1, the genomic locus where CCR5 is located contains multiple genes, such as transgenic dysregulated cancer-related genes that may be activated [160, 161].

#### Human ROSA26

ROSA26 refers to a locus that is widely used for achieving generalized expression in mouse models [162]. This locus has become a standard locus for transgene insertion in mouse embryonic stem cells. Irion et al. identified the human ROSA26 locus via the chromosome 3 (position 3p25.3) homology [162]. According to a study, a red fluorescent protein (RFP) reporter gene without a promoter integrated into this locus was consequently expressed in the cells of all three germ layers [162]. No further studies have yet assessed the suitability or safety of the human ROSA26 locus. As in the case of the above two loci, the human ROSA26 locus is also located near genes that may be potentially dysregulated by transgene integration into this locus [163]. However, the mouse homologous Rosa26 position is a “safe harbor” which permits the CAR transgene to be expressed safely by a targeted integration using genome-engineering techniques [164].

## Other considerable aspects

### CAR expression level and tonic signaling

Another key inspection for a successful CAR T cell design is the transgene regulation after its introduction into the T/NK cell genome. Generally, this aspect of a CAR design is strongly influenced by regulatory elements in the site of the insertion as well as the type of the used vector [165]. Gene expression is normally driven by enhancer and promoter regions in the retroviral LTR of the vector following retroviral transduction [165]; however, the most popular self-inactivation design in lentiviral vectors employs internal promoters to drive transgene expression [166]. In addition, the copy number of the used vector (insertion frequency) integrated into the genome may affect the transgene expression level [167]. The frequency of insertion is strongly influenced by the multiplicity of infection (MOI) used in the production procedure and is consequently a possible variable parameter [18, 168].

The level of CAR expression is an important factor that could result in insufficient receptor density which might reduce the sensitivity of CAR T cells to tumor cells that express low levels of antigen [169]. In addition to the main role of CAR expression, recent studies have also highlighted the important link between stimulatory domains and transgenic promoter selection [170]. Studies have shown that disrupting the TRAC gene by integrating a CD19-redirected CAR transgene in it can also improve the effectiveness of the developed CAR T cells [51]. The researchers have further demonstrated that integrating the CAR transgene into the TRAC locus can prevent tonic signaling and establish successful internalizing and re-expressing of CAR molecules, regardless of the CAR exposure to CD19; thereby delaying the differentiation of the effector T cells [25].

According to another study, to further define the importance of CAR expression levels, researchers generated T cells that express CAR from different genomic sites and promoters [25]. TRAC-EF1α CAR T cells, B2M-CAR T cells, TRAC-LTR CAR T cells, and TRAC-CAR T cells were developed and their efficacy was assessed in vitro and in vivo accordingly [25]. After repeated antigen stimulation, TRAC-EF1α CAR T cells quickly acquired an effector phenotype, while B2M-CAR T cells and TRAC-CAR T cells retained their central memory phenotype [25]. The down-regulation and subsequent re-expression of CAR protein are somehow similar to the regulatory role of TCR after stimulating antigen-induced TCR recycling in human and mouse T cells [25]. Together, these results highlighted the importance of CAR transgene integration and further indicated that the regulation of CAR expression is beyond the baseline transcriptional control [25]. Therefore, prevention of tonic signaling in the absence of antigen could promote optimal baseline expression, which can allow single or multiple CARs to be effectively internalized upon contact with an antigen [25]. The other factor is to direct a balanced transcriptional response, which recovers the kinetics of the baseline CAR expression after antigen encounter [25]. It was demonstrated that CAR T cells that have a CAR transgene under the TRAC regulatory elements lead to a better eradication of tumors, in contrast with CAR T cells with higher CAR expression levels [25]. Although, the endogenous B2M promoter has a response similar to the TRAC promoter after CAR stimulation, the in vivo performance of B2M-CAR T cells is not the same as the TRAC-CAR T cells; this might be due to the low basic CAR expression level that is not sufficient to effectively exert the antitumor activity of the CAR T cells. Ultimately, it is concluded that basic and dynamic CAR expression levels help maintain a more favorable T cell function [25].

### Three-dimensional nuclear organization, epigenetic marks, and regulatory DNA

Previously, choosing an appropriate insertion site for a CAR transgene only entailed one criterion; namely, kilobase-level genomic interactions (involving enhancer-promoter interactions) [171]. In fact, all known insertional oncogenesis incidents have occurred on this scale, so far. As understanding of the dynamic folding and packaging of the genome in the nucleus develops, we may become more aware of the impact of additional levels of genome organization and chromatin structure on transgene expression [172, 173]. Genome-wide studies using the capture of chromosome structures-based methods (such as 3C, 5C, and Hi-C) have shown that most genomic DNA is divided into topologically related domains (TAD) that comprises megabases in length. Genetic elements usually interact with each other within a domain, but rarely participate in inter-domain interactions. "*Non-loop*" DNA stretching between two TADs are originally known as TAD border region, and they prevent interaction between adjacent TADs [174]. Therefore, TAD is considered to represent a regulatory genomic unit in which enhancers and promoters can interact with each other [175, 176]. There is information about the relationship between chromatin topology and genomic activities. Genomic folding is not as firm and steady as protein structures, and whether this causes or influences a particular genome behavior is still unclear. However, there is clearly a relationship between the chromatin 3D structure and gene activity. In addition, TADs and their boundaries are mainly preserved among diverse cell types. Therefore, the virtual location of a genomic locus relative to the TAD could help us choose a GSH. It might be suitable to fully avoid a cancer-related gene site within a TAD or prefer a GSH at the border of a TAD [177].

Another layer of complexity is added through epigenetic modifications, which can be recorded, signaled, or permanently maintained in the active state of the genome [178]. These comprise DNA alterations, such as 5-methylcytosine and 5-hydroxymethylcytosine, histone modifications, and nucleosome remodeling. The accessible, transcriptionally active, and inhibitory chromatin domains are distinguishable [179]. These characteristics may help predict the ability of a given genomic locus to support sufficient CAR transgene expression. Moreover, the nucleosomes near the enhancer usually hold histones with characteristic tail modifications at the amino terminus; for instance, the histone H3 lysine 4 monomethylation and H3K27 acetylation showed that the polycomb protein-related repressor is enriched with H3K27me3 markers [180]. Compared with the contribution of epigenetic characteristics to gene insertion-related diseases, DNA sequencing can easily be used to analyze the linear organization of the genome where insertional mutagenesis occurs in preclinical and clinical models [181]. An epigenetic investigation is a rather difficult task, since epigenetic modifications are dynamic, unstable, and cell-dependent features. Also, the integration of a given transgene might reshape the surrounding chromatin in ways that it is not yet fully understood [182].

**Fig. 3:**
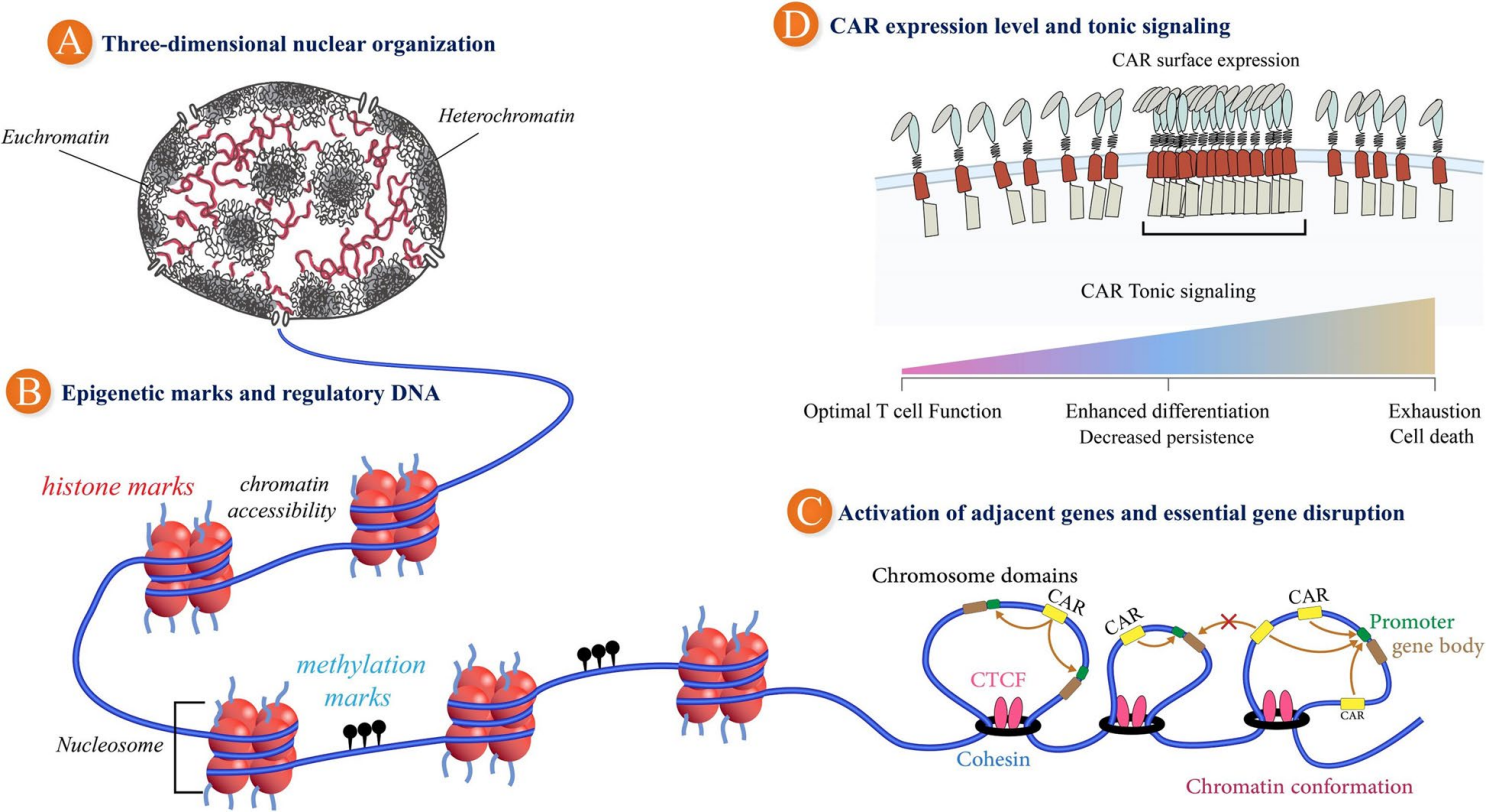
Principles and criteria for choosing appropriate safe harbors for CAR transgene integration into the human genome. **A** Three-dimensional nuclear organization. The DNA strands inside the cell nucleus are in the open form (Euchromatin) or compressed form (Heterochromatin). Inserting the CAR construct into the Heterochromatin regions will imping on its consequential expression. **B** Epigenetic markers and regulatory DNA. Another level of regulation of gene expression in the genome is through epigenetics. In this case, according to histone codes and DNA methylations, the structure of the nucleosomes is changed (chromatin remodeling) and the expression of a gene is allowed. If a CAR transgene is integrated into an active transcription region, it is more likely for the transgene to be expressed correctly. **C** Activation of adjacent genes and essential gene disruption. DNA strands inside the nucleus are organized into TAD regions. These areas are separated by insulator elements. If a CAR construct is positioned within a TAD region, it is possible that it might interact with the neighboring genes in the same region, but not with other genes outside that given TAD. **D** CAR expression level and tonic signaling. If the CAR transgene is expressed permanently without proper regulation, the CAR protein accumulates on the surface of the engineered cell as they also bind to each other, causing tonic signaling. Conversely, if the CAR transgene is adjusted by appropriate regulatory factors such as promoter, enhancer, and silencers, the expression is carried out dynamically when needed, the frequency of CAR protein on the cell surface is appropriate and does not cause tonic signaling. Tonic signaling leads to more T cell differentiation and exhaustion

It is generally believed that an integration into the "repressive" chromatin regions may lead to silencing, while a favorable transcriptional activity and also a transgene expression are attributed to the "active" chromatin regions, which are considered ideal in terms of appropriate GSHs [183]. Even though the data regarding the status of chromatin might help expression prediction from any certain genomic locus in any given T/NK cell phenotype, there is still insufficient information in terms of the epigenetic events [184, 185] (Fig. 3).

## Clinical trials and approved products

Today is a turning point in the evolution of a completely novel scientific paradigm for the treatment of serious diseases. In just a few decades, gene therapy has progressed from a promising concept to a viable treatment option for deadly and incurable cancers. *Yescarta* (*axicabtagene ciloleucel*), the second gene therapy approved by the US FDA, and *Kymriah* (*tisagenlecleucel*), a first-of-its-kind treatment modality for certain patients with B-cell malignancies, are both CAR T cell therapies [31]. *Tecartus* (*brexucabtagene autoleucel*), *Breyanzi* (*lisocabtagene maraleucel*), *Abecma* (*idecabtagene vicleucel*), and *Carvykti* (*ciltacabtagene autoleucel*) are other FDA-approved products developed over time [31].

Several clinical trials have been carried out based on the site-specific integration of CAR transgenes [186]. Currently, there is not sufficient clinical evidence to support whether a host T cell PD-1 immuno-editing is more beneficial and/or equal to the PD-1 mAb treatments. It seems that cell-intrinsic disruption of immune checkpoints genes via gene editing is likely to have a better safety profile than systemic administration of blocking mAbs [35].

Today, there are clinical trials evaluating patient treatment using PD-1-deficient CAR-T cells (NCT03747965 and NCT03545815), in Lung cancer (NCT03525782), refractory B-cell malignancy (NCT03298828 and ChiCTR1800020306), esophageal cancer (NCT03706326), prostate cancer (NCT03525652), and various other solid tumors. Only three of these registered trials have released their preliminary results [187–189]. According to one study, Lu and colleagues conducted the first-in-man clinical trial (NCT02793856) in patients with advanced non-small cell lung cancer (NSCLC) to assess the safety of a CRISPR/Cas9-mediated knockout of the PD-1 gene in autologous T lymphocytes [190]. They used an escalating dosage scheme for 11 patients [190]. The most common adverse effects were acute fever and hepatic dysfunction, according to the data collected from 8 patients who received 16 cycles of PD-1^Knockout^ T cell infusions and three patients in the control group [190]. There were no dose-limiting toxicities and/or other grade 3–5 adverse events, which may confirm the safety profile of the PD-1^Knockou^ T cells in these patients [190]. Lu and colleagues found evidence of potential responsive T cell clones in the patients’ peripheral bloods over the course of treatment using next-generation sequencing.

According to another investigation, PD-1^Knockout^ MUC1-redirected CAR T cells were found to have a low rate of adverse events in 8 enrolled patients with advanced NSCLC [189]. Of note, there were no grade 3–5 adverse events, indicating that the infused CAR T cell product was well-tolerated [189]. Patients who received the low-dose regimen had a moderate treatment response, according to preliminary data [189]. Another phase trial I investigating genome-edited T Cells (PBLTT52CAR19) in R/R B-ALL patients began in August 2020, with allogenic engineered human T cells (defined as TT52CAR19^+^ TCR^-^) for the treatment of CD19^+^ patients [191]. The cells were not HLA-matched and were from healthy adult donors, and they were transduced with CD19-redirected CARs using a lentiviral vector that also contained CRISPR guides for the genome editing of the CD52 and TRAC loci in the presence of Cas9 [191]. Of note, patients who achieve molecular remission will be eligible for an allo-HSCT.

## Conclusions

Undoubtedly, applying CAR T cells is considered as one of the most fruitful therapeutic approaches for the treatment of blood-based cancers. The clinical success of this treatment modality in blood malignancies led to the US FDA approval of six CAR T cell products, and considering the wide range of antigen specificity, CAR-based therapies might have a major potency for the treatment of various other oncological indications [4, 34]. Despite all the above discussions, it is safe to conclude that improving the performance of CAR T cells can have a promising future in both research and therapeutic aspects [192].

In summary, we reviewed the application of site-specific integration of CAR transgenes in terms of clinical applicability and CAR T cell phenotype. It has been demonstrated that integrating a CAR-encoding sequence into the TCR locus and placing it under the control of the endogenous regulatory elements reduces tonic signaling, averts an accelerated T cell differentiation and exhaustion, and increases the therapeutic potency of the engineered CAR T cells [25, 51]. The kinetic measurement of antigen-induced CAR internalization and degradation demonstrates that CAR expression and variations of cell surface CAR is dependent on enhancer/promoter elements [25]. These findings indicate that a strict transcriptional regulation of CAR expression is essential for an effective tumor eradication. Therefore, CAR transgene integration into the TCR locus, which minimizes the risks of insertional oncogenesis and TCR-induced autoimmunity and alloreactivity, can lead to a safer CAR T cell therapy. Eventually, by reducing constitutive signaling and delaying T cell exhaustion, a more potent CAR T cell product can be achieved. It seems that there is a correlation between CAR immunobiology and the potential of genome-editing technologies for the development of safer and more effective CAR-based immunotherapeutics.

## Data Availability

Not applicable.

## Abbreviations

CARs: Chimeric antigen receptors
TCR: T-cell receptor
mAbs: Monoclonal antibodies
NK: Natural killer
FDA: Food and Drug Administration
BCMA: B-cell maturation antigen
MLVs: Murine leukemia viruses
SB: Sleeping Beauty transposons
DSBs: Double-strand breaks
rAAV: Recombinant Adeno-Associated Viruses
ZFNs: Zinc finger nucleases
TALENs: Transcription activator-like effector nucleases
AAV: Adeno-Associated Viruses
RV: Retroviruses
LV: Lentiviruses
GSH: Genomic safe harbor
GvHD: Graft-versus-host disease
GMP: Good manufacturing practice
TME: Tumor microenvironment
PD-1: Programmed cell death-1
CTLA-4: Cytotoxic T-lymphocyte antigen 4
TNF-α: Tumor necrosis factor α
AICD: *activation-induced cell death*
KO: Knockout; Th, T-helper
NHEJ: Non-homologous end joining
HDR: Homologous direct repair
AAVS1: AAV Site 1
ITR: Inverted terminal repeats
AAV6: AAV serotype 6
CTLs: Cytolytic T cells
GBM: Glioblastoma multiform
RVD: Repeated variable di-residue
HE: Homing endonuclease
LMO2: LIM domain protein 2
CCND2: Cyclin D2
ncRNAs: Non-coding RNAs
lncRNA: Long non-coding RNA
TAD: Topologically related domains
CCR5: Chemokine (C–C motif) receptor 5
RFP: Red fluorescent protein
B-ALL: B-cell acute lymphoblastic leukemia
